# Electroacupuncture in non-surgical management of lumbar spinal stenosis: mechanistic potential in attenuating ligamentum flavum thickening via inflammatory factor modulation

**DOI:** 10.3389/fimmu.2025.1644394

**Published:** 2025-10-30

**Authors:** Hao-Xin Shi, Yu-Jun Gao, Shu-Ren Wang

**Affiliations:** ^1^ Graduate School, Heilongjiang University of Chinese Medicine, Harbin, China; ^2^ Department of Traditional Orthopedic Therapy, The First Affiliated Hospital of Heilongjiang University of Chinese Medicine, Harbin, China

**Keywords:** lumbar spinal stenosis, ligamentum flavum thickening, electroacupuncture, inflammation, signal pathway

## Abstract

Lumbar Spinal Stenosis (LSS) is a prevalent spinal disorder mainly induced by degenerative changes in the spine, which lead to nerve root compression. This results in symptoms such as lower back pain, numbness of the lower limbs, and difficulty in walking. The thickening of the ligamentum flavum (LF) is a crucial pathological feature of LSS and is closely linked to inflammatory responses. Electroacupuncture (EA), a form of traditional Chinese medical therapy, has garnered increasing recognition in modern medicine in recent years. It has shown notable efficacy in alleviating pain and enhancing function. EA achieves these effects by modulating inflammatory cytokines, reducing pro-inflammatory markers such as tumor necrosis factor-alpha (TNF-α), interleukin-1 beta (IL-1le, and interleukin-6 (IL-6), while increasing anti-inflammatory cytokines like interleukin-10 (IL-10). Additionally, EA may inhibit LF thickening by suppressing signaling pathways, specifically the nuclear factor-κB (NF-κB) pathway, the Janus kinase/signal transducer and activator of transcription (JAK/STAT) pathway, and the mitogen-activated protein kinase (MAPK) pathway. Clinical studies indicate that when EA is combined with other treatment modalities, it can significantly reduce pain and improve functional status in patients with LSS, thus enhancing their quality of life. Although the mechanisms underlying the effects of EA in the treatment of LSS warrant further exploration, its ability to regulate inflammatory responses through multiple pathways and promote tissue repair provides new perspectives and directions for the non-surgical management of LSS. This review encapsulates the application of EA in LSS and explores its potential mechanisms in mitigating LF thickening through the modulation of inflammatory cytokines. The aim is to offer a reference for future research and clinical practice.

## Introduction

**Figure 1:**
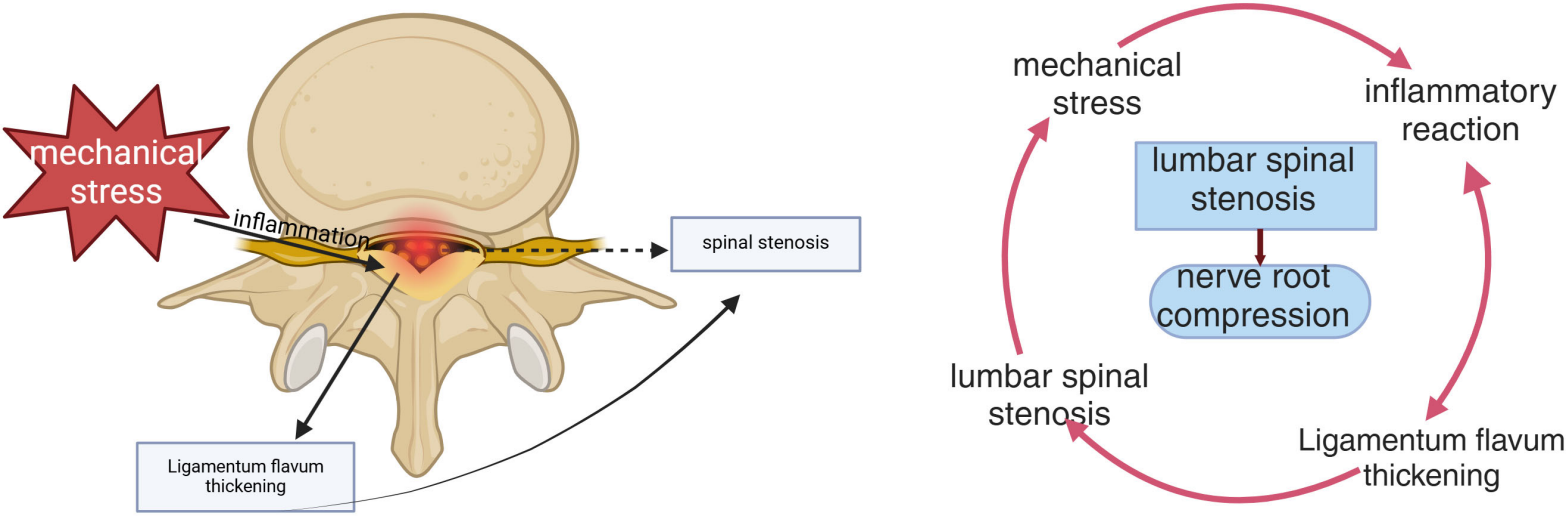
LF thickening-induced spinal canal stenosis. Mechanical stress initiates the thickening of the LF, causing tissue damage and inflammatory responses. This inflammation further contributes to the accumulation of scar tissue and fosters the ongoing thickening of the LF through inflammatory cytokines. Ultimately, this process compresses the nerve roots and leads to the development of LSS.

Lumbar spinal stenosis (LSS) is typically attributed to degenerative changes within the spine, which may alter its anatomical structure and normal physiological angle, thus compressing the nerve roots (1). The primary symptoms of LSS include lower back pain, numbness and weakness in the lower limbs, and difficulty walking, with a notably higher prevalence among older adults. Epidemiological studies reveal that LSS affects approximately 103 million people worldwide, with its prevalence rising significantly with age. Imaging-based diagnostic studies suggest that the prevalence of asymptomatic LSS is around 11%, climbing to as much as 38% within the general population. LSS is relatively uncommon in individuals under 50 years of age. However, as aging progresses and lifestyle habits shift, its incidence is expected to rise. These findings highlight LSS as a significant public health concern, with its impact particularly severe on the elderly population (2, 3). The development of LSS is closely linked to degenerative factors such as intervertebral disc protrusion, facet joint osteophyte formation, and ligamentum flavum (LF) thickening, with LF thickening considered a crucial contributing factor (4). The LF is attached to the posterior and lateral walls of the spinal canal. Thickening of the LF reduces the diameter of the spinal canal, compressing the dural sac and nerve roots, potentially leading to symptoms like neurogenic claudication. As aging occurs, the LF undergoes fibrotic changes and thickening due to the cumulative effects of mechanical stress. Research indicates that mechanical stress is the primary factor behind LF thickening. It activates fibroblasts in the LF, triggering an inflammatory response and releasing various inflammatory mediators, such as transforming growth factor-beta (TGF-β) and other pro-fibrotic factors. These mediators further encourage cell proliferation and excessive extracellular matrix(ECM) deposition, ultimately resulting in the thickening and fibrosis of the LF (5). Consequently, LF thickening reduces the space and volume of the spinal canal, exacerbating the severity of LSS and further worsening the symptoms experienced by patients (**Figure 1**)

Throughout history, traditional Chinese medicine (TCM) therapies ranging from Gua Sha to chiropractic manipulation have utilized mechanical stimulation to influence internal physiological environments for their analgesic and anti-inflammatory effects. For example, Gua Sha is recognized for initiating immune responses by generating local petechiae (6), and clinical observations have documented the spontaneous resolution of lumbar disc herniation following conservative management strategies, including spinal manipulation (7, 8). In recent years, electroacupuncture (EA) has gained significant prominence in modern medicine as an enhanced therapeutic approach. EA is grounded in classical acupuncture principles dating back over two millennia. Since the 1950s, the integration of controlled electrical parameters has enabled EA to combine needling with electrical stimulation (9), effectively modulating physiological functions, alleviating pain, and regulating inflammatory responses (9). Studies have shown that EA reduces the expression of inflammation-related cytokines, such as tumor necrosis factor-alpha (TNF-α),interleukin-1 beta (IL-1α), and interleukin-6 (IL-6), while increasing anti-inflammatory cytokines like interleukin-10 (IL-10), resulting in substantial therapeutic effects (10). In treating LSS, EA demonstrates considerable clinical value, especially in pain relief and functional improvement. The primary treatment options for LSS include pharmacological therapy, physical therapy, acupuncture therapy and surgical intervention. However, pharmacological treatments often lead to side effects, such as gastrointestinal discomfort and liver or kidney damage, with long-term use potentially causing dependency. While physical therapy can relieve symptoms, it usually offers limited effects and requires consistent patient adherence. Surgical intervention, though effective in relieving nerve compression, presents higher risks, involves extended recovery periods, and is not suitable for all patients. As a simple and convenient therapeutic approach, EA offers a safe and effective alternative for LSS patients, exhibiting significant advantages in the non-surgical management of this condition. Toroski et al. (11) demonstrated that EA is a dominant strategy for chronic low back pain, yielding a superior utility score (0.70 vs. 0.63) at a lower annual cost ($461.48 vs. $497.77) compared to nonsteroidal anti-inflammatory drugs (NSAIDs). In a study with 17 patients who did not respond to conventional conservative treatments or traditional acupuncture (12), spinal nerve root EA significantly improved lower back pain, lower limb pain, and sensory abnormalities (visual analog scale(VAS) scores: back pain, p < 0.05; lower limb symptoms, p < 0.01), with therapeutic effects lasting up to three months.

Although EA has shown considerable effectiveness in relieving pain and enhancing function, its potential mechanisms in reducing LF thickening through the regulation of inflammatory cytokines remain insufficiently examined. Therefore, this review aims to explore the use of EA in LSS, with a particular emphasis on its potential mechanisms for improving LF thickening by modulating inflammatory cytokines. By conducting a thorough analysis of the mechanisms behind EA, this study seeks to offer new insights into the treatment of LSS and foster the integration of traditional Chinese medicine with modern medical practices.

## Methodology

We initiated the literature search by screening titles. For those that appeared relevant, we then read through their abstracts and conclusions to decide which ones to subject to a more detailed assessment. This step narrowed down our list significantly. The final step involved a thorough, full-text review of these shortlisted articles to determine which ones would ultimately form the core literature for our review.

To make sure we didn’t miss any important studies, we also went through the reference lists of our final set of articles, picking out any that seemed connected to our research question. We also added a few other papers that served as good examples or helped illustrate specific methods and points.

All searches were conducted using three main databases: Web of Science, PubMed, CNKI, and Wanfang. We used a range of search terms to ensure comprehensive coverage, including “lumbar spinal stenosis”, “electroacupuncture”, “ligamentum flavum”, “hypertrophy”, “inflammation”, “inflammatory cytokines”, and key signaling pathways such as the NF-κB pathway, JAK/STAT pathway, and MAPK pathway.” We made sure to use the Chinese equivalents of all these terms in the Chinese databases.

## LSS

### Pathological mechanisms of LSS

The etiology of LSS is categorized into primary and secondary causes. Primary LSS stems from congenital abnormalities or developmental disorders, while secondary LSS primarily arises from degenerative changes. These changes include decreased intervertebral disc height, facet joint hypertrophy, LF thickening, and spondylolisthesis (1, 3). LSS impacts several anatomical regions: the central canal, lateral recess, and intervertebral foramen. Central canal stenosis results from reduced disc height, disc bulging, facet joint hypertrophy, and LF thickening. LSS is characterized by its dynamic nature; the spinal canal’s space fluctuates with different postures—decreasing during weight-bearing and extension, and increasing during flexion and axial traction. These dynamic changes significantly influence the clinical presentation of LSS symptoms (1). Additionally, LSS can induce nerve root compression, leading to impaired blood flow, occlusion of small arteries, neural ischemia, and diminished nutrient supply to the nerve roots, clinically manifesting as pain, numbness, and weakness (13). Furthermore, stenosis can obstruct venous return, elevate venous pressure, and cause the accumulation of toxic metabolites, which further exacerbates nerve root damage (3).

### Pathological mechanisms of LF thickening

Inflammatory responses in joints and the LF, particularly the elevation of inflammatory cytokines, are closely linked to the development of LSS. The inflammatory process in LSS is primarily associated with pathological changes of the LF. Studies have demonstrated a strong correlation between LF thickening and the accumulation of scar tissue induced by inflammation. In thickened LF, fibrosis (scar formation) is prominent, with inflammation serving as the catalyst for this process. The thickening of the LF could result from tissue damage caused by mechanical stress, which triggers inflammatory responses and the subsequent accumulation of scar tissue. Research has shown that, in patients with LSS, the LF undergoes fibrosis and loss of elastic fibers, which are associated with the activation of inflammatory cytokines. Within the inflammatory milieu, cytokine release stimulates surrounding cells to synthesize fibrous matrix components, thereby initiating a process of fibrosis that culminates in increased LF stiffness (14). Various inflammation-related cytokines are expressed in the LF, including cyclooxygenase-2 (COX-2), TNF-α, and several interleukins such as interleukin-1 (IL-1), IL-6, interleukin-8 (IL-8), and interleukin-15 (IL-15). COX-2, an enzyme linked to inflammation, has been detected in thickened LF, underscoring the critical role inflammation plays in LF thickening. Cytokines such as IL-1β promote the production of matrix metalloproteinases (MMPs), leading to excessive degradation and remodeling of the ECM, thus accelerating LF thickening (15). MMPs, a class of enzymes involved in ECM degradation, when abnormally expressed, can disrupt tissue structural balance. A recent clinical study (16) on human LF tissue confirmed that IL-6 acts as a critical upstream signal driving MMP-mediated elastic fiber degradation. The research demonstrated that both IL-6 and MMP-2 were significantly upregulated in hypertrophied LF from patients with LSCS, and their levels exhibited a strong positive correlation. Mechanistically, IL-6 directly induces the expression, secretion, and activation of MMP-2 in LF fibroblasts by activating the signal transducer and activator of transcription 3 (STAT3) pathway. Functional assays confirmed that this process ultimately accelerates elastolysis, thereby disrupting the normal elastic fiber architecture of the LF and contributing to its pathological thickening characterized by elastic fiber loss and collagen disorganization. Studies have indicated that chronic inflammation is a significant factor contributing to LF degeneration and thickening in LSS (15–19). Chronic inflammation contributes to LF thickening not only by facilitating ECM degradation but also by directly driving pathological fibrotic deposition. Inflammatory mediators such as IL-6 and TNF-at originating from adjacent degenerative discs, can directly stimulate LF cells to initiate a profibrotic response. Park et al. (20) demonstrated that this response involves a significant upregulation of collagen types I, V, and XI mRNA, promoting aberrant ECM accumulation. Importantly, a recent study employing a novel rabbit model (21) delineated that this inflammatory cascade is initiated by mechanical stress. The stress-induced microenvironment, characterized by M1 macrophage infiltration and the release of pro-inflammatory factors (TNF-α, IL-6), markedly enhances the secretion of transforming growth factor-beta 1 (TGF-β1) from LF fibroblasts. TGF-β1, in turn, acts as a key driver for the transition of fibroblasts into α-smooth muscle actin (α-SMA)-expressing myofibroblasts, culminating in excessive synthesis of collagens (e.g., types I and III), aberrant ECM deposition, and ultimately, fibrotic hypertrophy of the LF.

Notably, while IL-6 has been identified as a key mediator in this inflammatory-fibrotic cascade, the upstream regulatory mechanisms that control IL-6 expression in hypertrophic LF remain an important area of exploration. Recent research (19) has uncovered a critical role of angiopoietin-like protein 2 (Angptl2) in this process—one that links inflammation, angiogenesis, and LF thickening by modulating IL-6 signaling. These investigations further indicate that Angptl2, which regulates inflammation and angiogenesis, is highly expressed in hypertrophic LF. Angptl2 promotes inflammatory responses by activating IL-6 expression. The expression of IL-6 is significantly elevated in hypertrophic LF tissue and positively correlates with LF thickness and Angptl2 levels. Angptl2 binds to integrin α5β1, a cell surface receptor involved in ECM signaling on LF fibroblasts, activating the NF-κB signaling pathway. This activation induces the expression and secretion of IL-6. These findings highlight the intimate relationship between inflammation and LF degeneration and thickening, underscoring the critical role of inflammatory responses in the pathological mechanisms of LSS.

The release of inflammatory mediators not only worsens tissue degeneration but also heightens pain sensitivity by affecting nerve roots. Research indicates that the buildup of macrophages is linked with the release of inflammatory cytokines, which promote inflammation and edema of the nerve roots, thus intensifying pain. Therefore, the release of inflammatory mediators plays a crucial role in the pathological processes following spinal cord injury (22). Inflammation also contributes to the formation of localized edema in neural structures, further narrowing the spinal canal and nerve root pathways, thereby worsening nerve compression symptoms. This results in clinical manifestations such as lower back pain, radicular pain, and cauda equina syndrome (15). Consequently, controlling the inflammatory response is vital for alleviating symptoms associated with LF thickening and for decelerating disease progression.

## Multiple mechanisms of EA in the treatment of LSS

EA demonstrates various therapeutic mechanisms in treating LSS, including analgesia, neuromodulation, enhancement of microcirculation, as well as regulation of immune and inflammatory responses. By promoting the release of endogenous opioids, EA can significantly alleviate pain caused by nerve root compression (23). Additionally, EA modulates neurotransmitter release and improves neuronal function, thereby reducing pain (10). In improving microcirculation, EA stimulates specific acupoints to increase local blood flow and microvascular density, alleviating nerve compression, reducing pain, and facilitating functional recovery (24, 25). Furthermore, EA activates local immune responses, regulates macrophage polarization, and balances the release of pro-inflammatory and anti-inflammatory factors. Through neural regulation, EA mitigates inflammation, promotes tissue repair, and maintains systemic immune homeostasis (26).

## Mechanisms of EA in regulating inflammatory responses

### The role of inflammatory factors in the pathogenesis of LSS

**Figure 2:**
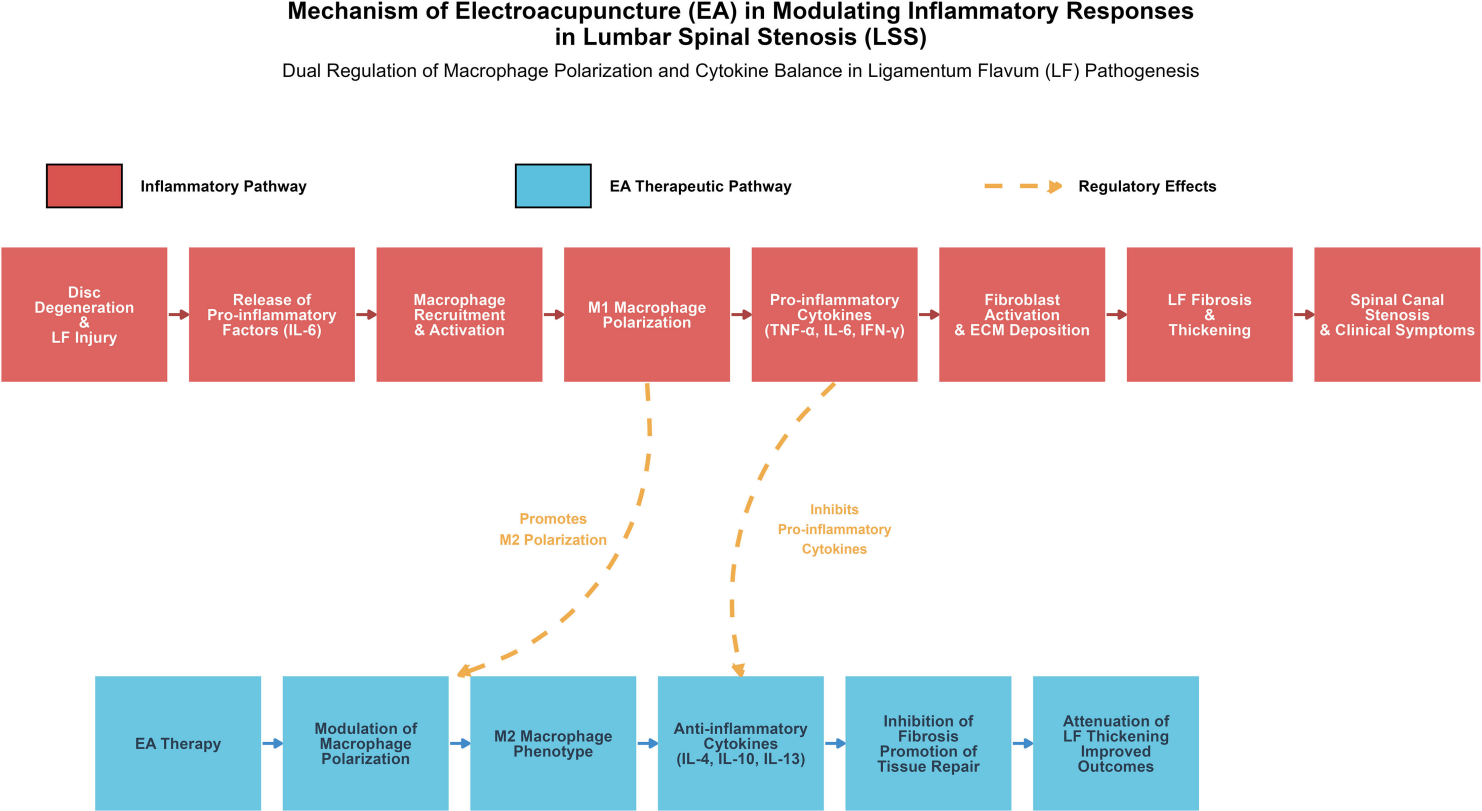
Mechanism of EA in modulating inflammatory responses and macrophage polarization in LSS. In the pathogenesis of LSS, LF injury triggers an early inflammatory response, leading to macrophage recruitment and polarization toward the M1 phenotype. M1 macrophages release pro-inflammatory cytokines (e.g., TNF-α, IL-6, IFN-γ), which activate fibroblasts and promote ECM deposition, resulting in LF fibrosis and thickening. These changes contribute to spinal canal stenosis and clinical symptoms. EA therapy modulates macrophage polarization by suppressing the M1 phenotype and promoting the M2 phenotype, which secretes anti-inflammatory cytokines (e.g., IL-4, IL-10, IL-13). This shift inhibits fibrotic processes, facilitates tissue repair, ameliorates LF thickening, and improves clinical outcomes.

LSS is often associated with intervertebral disc degeneration, LF thickening, and other structural changes in the spine, which incite localized inflammatory responses. Research has demonstrated that the irregular expression of inflammatory factors plays a crucial role in the onset, progression, and prognosis of LSS. Modulating these inflammatory responses by decreasing the release of pro-inflammatory factors and fostering the production of anti-inflammatory factors can effectively prevent or control the advancement of LSS (27). Inflammatory factors and immune cells are pivotal in the pathological processes of LSS. Damaged areas attract immune cells like macrophages, which manage local inflammatory responses by secreting mediators such as cytokines and chemokines. In the development of LF thickening, inflammatory factors are key drivers. LF injury triggers an early inflammatory response, significantly increasing pro-inflammatory factors like IL-6. As inflammation persists, macrophages invade the damaged tissue, and pro-inflammatory factors intensify the inflammatory reaction, leading to fibrosis, structural changes, reduced elasticity, increased stiffness, and LF thickening. This fibrosis further exacerbates spinal canal stenosis and contributes to related clinical symptoms (28). Conversely, anti-inflammatory factors like IL-10 can mitigate inflammatory responses and promote tissue repair, thereby alleviating LF fibrosis and thickening (19). This mechanism highlights the central role of the inflammatory state in LF’s pathological changes, suggesting that factors regulating inflammatory responses may serve as potential therapeutic targets for LSS. Macrophages are critical in the recovery process following spinal cord injury and are key effector cells in response to spinal injury. Under inflammatory conditions, macrophages often polarize toward the M1 phenotype, releasing pro-inflammatory factors such as TNF-α, IL-6, and interferon-γ (IFN-γ), which contribute to chronic low-grade inflammation and delay tissue repair. In contrast, M2 macrophages secrete anti-inflammatory factors like interleukin-4 (IL-4) and IL-10, which help suppress inflammation and promote tissue repair. EA therapy has been shown to influence macrophage polarization by inhibiting the pro-inflammatory effects of M1 macrophages and promoting their polarization toward the M2 phenotype, thus alleviating inflammation and accelerating tissue repair (14, 29). Mingyang Yan et al. (30) and colleagues validated this mechanism using a rat model of skeletal muscle contusion-induced fibrosis. Their study showed that EA significantly reduced M1 macrophages and their markers, such as IFN-γ, while increasing M2 macrophages and their markers, such as IL-4 and interleukin-13 (IL-13). These findings suggest that EA effectively modulates inflammatory responses and supports the repair and regeneration of injured skeletal muscle by facilitating macrophage polarization. Moreover, EA alleviates fibrosis and enhances the regeneration of muscle fibers. Based on these findings, EA may hold therapeutic potential for LSS, particularly in the pathology of LF thickening, which is closely linked to fibrosis. By modulating macrophage polarization, EA could inhibit fibrotic responses and improve the pathological changes caused by LF thickening, offering a novel approach to treating LSS. Through phagocytosis and the secretion of degradative enzymes, macrophages can help clear damaged tissue, aiding in restoring functionality in the affected area (22, 27, 31). While the inflammatory response contributes to tissue repair, it may also exacerbate pain. Inflammatory mediators can stimulate nerve fibers, leading to a reduced pain threshold and spontaneous pain occurrence (10, 31). Thus, inflammation in LSS patients may not only be part of the pathological process but also a direct cause of symptom variation (see **Figure 2**)

### EA regulates inflammatory response

**Figure 3:**
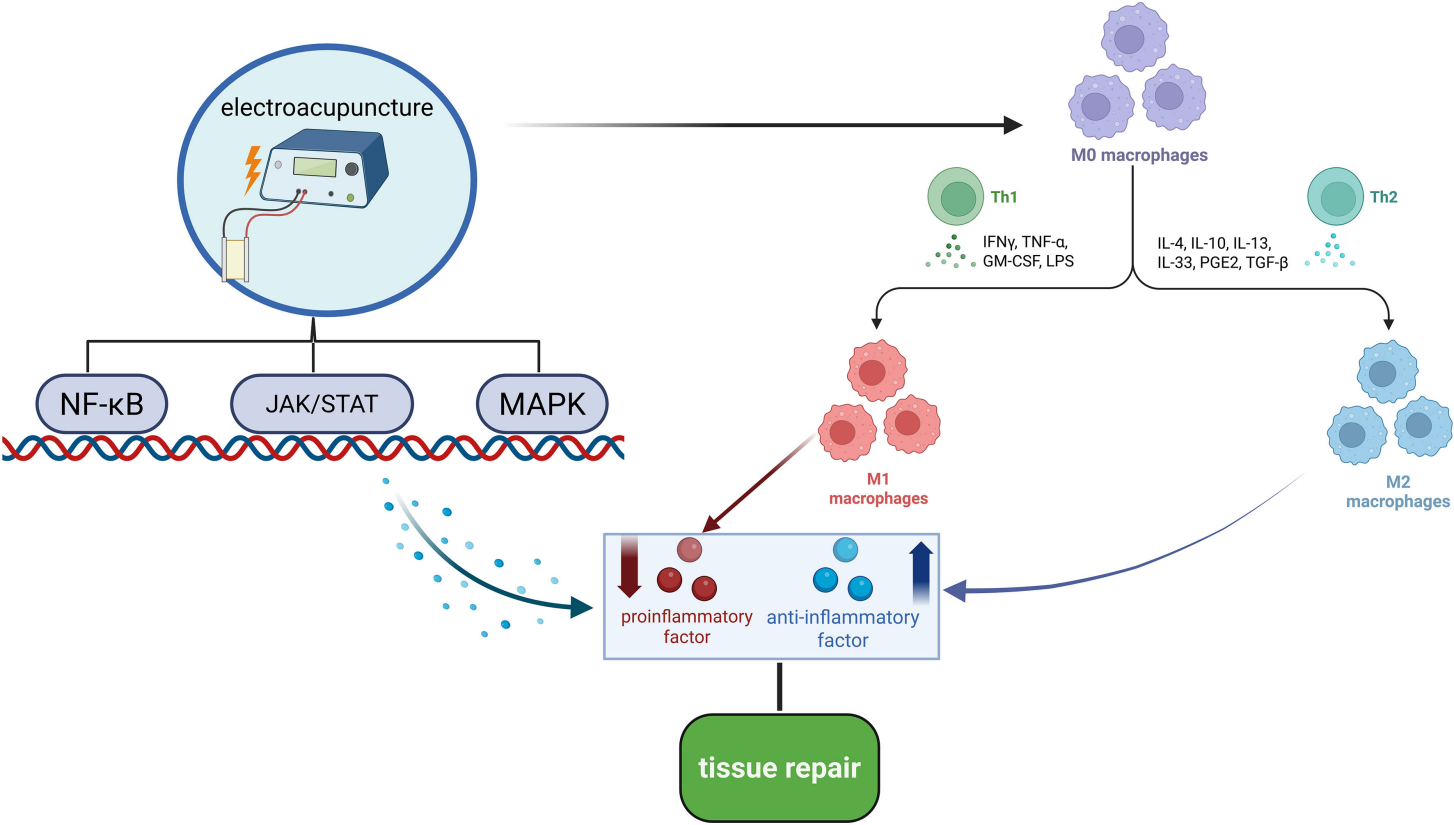
Mechanism diagram of EA regulating inflammatory factors. EA alleviates local inflammatory responses and promotes tissue repair by activating the NF-κB, JAK/STAT, and MAPK signaling pathways, which downregulate the expression of pro-inflammatory factors while upregulating the expression of anti-inflammatory factors. Additionally, EA modulates macrophage polarization by inhibiting their polarization toward the pro-inflammatory M1 phenotype and promoting their polarization toward the anti-inflammatory M2 phenotype, further reducing inflammation and facilitating tissue repair. Through these mechanisms, EA ultimately mitigates the pathological thickening of the LF and alleviates the symptoms of LSS.

EA has demonstrated considerable effectiveness in treating inflammation (31–34), particularly in alleviating pain associated with inflammatory processes. Its analgesic effects are primarily achieved through the modulation of various inflammatory factors. EA mitigates inflammatory responses by activating bioactive substances within the body and inhibiting the release of pro-inflammatory cytokines. This process involves EA’s regulation of immune cells, which promotes the expression of anti-inflammatory factors, thus improving pain and inflammatory conditions (23). Specifically, EA reduces levels of pro-inflammatory cytokines, such as TNF-α, IL-1β, and IL-6, while enhancing the expression of anti-inflammatory cytokines like IL-10. This regulatory effect is primarily mediated through its influence on immune cells, including macrophages and regulatory T cells (10, 29, 35) (see **Figure 3**). Additionally, EA can activate the hypothalamic-pituitary-adrenal (HPA) axis, encouraging the secretion of cortisol from the adrenal glands to suppress inflammatory responses. The HPA axis is a crucial regulatory system for the body’s response to stress and inflammation. When the body encounters pain or inflammatory stimuli, the HPA axis is activated, causing the hypothalamus to release adrenocorticotropic hormone (ACTH), which subsequently stimulates the adrenal glands to secrete cortisol. As a key anti-inflammatory hormone, cortisol effectively suppresses inflammation and aids in restoring homeostasis (33).

Studies indicate that EA can relieve pain caused by LSS through various mechanisms, including inducing neurogenic inflammation, releasing endogenous substances, and activating anti-inflammatory cascades. These processes ultimately lead to the release of endogenous opioids (36). Furthermore, in an animal study, EA stimulation at the Zusanli (ST36) acupoint was found to reduce paw swelling caused by delayed-type hypersensitivity (DTH), inhibit inflammatory cell infiltration, and lower the levels of specific immunoglobulins IgG and IgE. EA also helps regulate the Th1/Th2 balance by reducing the production of Th1-type cytokines, such as IFN-γ and TNF-α. Th1 cells are responsible for pro-inflammatory responses, while Th2 cells are involved in anti-inflammatory processes. By balancing these responses, EA effectively mitigates allergic and inflammatory conditions (37). Additionally, in a rabbit model of intervertebral disc degeneration induced by axial compression, EA applied at the lumbar “Jiaji” acupoints (EX-B2) exhibited notable anti-inflammatory effects. Experimental results demonstrated that EA significantly downregulated the expression of pro-inflammatory cytokines TNF-α and IL-1β in nucleus pulposus tissue, while also suppressing the secretion of senescence-associated secretory phenotype (SASP) factors such as IL-6 and IL-8 (38). These findings suggest that EA, as a non-surgical treatment modality, may provide a potential therapeutic approach for mitigating LF thickening in LSS by modulating inflammatory factors.

## Signaling pathways of EA in inflammation regulation

The classical inflammatory signaling pathways include the nuclear factor-κB (NF-κB) pathway, the Janus kinase/signal transducer and activator of transcription (JAK/STAT) pathway, and the mitogen-activated protein kinase (MAPK) pathway. The activation and regulation of these pathways directly influence the intensity and duration of inflammatory responses.

### NF-κB pathway

The NF-κB pathway is a pivotal signaling mechanism crucial for the initiation and regulation of inflammatory responses. By activating pro-inflammatory cytokines such as IL-1 and TNF-α, NF-κB governs the expression of a suite of pro-inflammatory genes, including cytokines, chemokines, and adhesion molecules. This modulation of immune cell functions amplifies the inflammatory response. Furthermore, NF-κB enhances cell survival by suppressing apoptosis-related gene expression, enabling cells to endure stress. Notably, NF-κB has a complex dual role: it promotes both inflammatory responses and, under certain conditions, anti-inflammatory processes that resolve inflammation (34, 39–41). Studies indicate that EA can inhibit the excessive activation of the NF-κB signaling pathway. EA significantly diminished the expression of pro-inflammatory cytokines such as TNF-α and IL-1β, while modulating anti-inflammatory cytokines like IL-10, thereby exerting anti-inflammatory effects (35). Importantly, pro-inflammatory cytokines like TNF-α and IL-1β, along with IL-10, activate downstream signaling pathways, culminating in the activation of the IκB kinase (IKK) complex. This complex primarily comprises IKKα, IKKβ, and IKKγ (NEMO). IKKβ phosphorylates inhibitor of κB (IκB) proteins, which bind to NF-κB, preventing its translocation from the cytoplasm to the nucleus. The degradation of IκB releases NF-κB, enabling it to enter the nucleus and activate the transcription of target genes (41–43). This intricate regulatory pathway drives the persistence and amplification of inflammatory responses. WU et al. (42) demonstrated that EA effectively downregulates inflammatory factors IL-1β, IL-6, and TNF-α in a knee osteoarthritis model, mitigating cartilage degeneration by modulating the NF-κB signaling pathway. Specifically, EA regulates the inflammatory response by inhibiting IKK-β and NF-κB p65 expression while upregulating IκB-α levels, thereby protecting articular cartilage and improving joint function. These findings provide vital molecular evidence supporting the use of EA in treating osteoarthritis, highlighting its potential efficacy in reducing inflammation and preserving joint tissue. In addition, Xia et al. (44, 45) demonstrated that 2 Hz EA significantly suppressed the expression of high mobility group box 1 (HMGB1), Toll-like receptor 4 (TLR4), myeloid differentiation primary response 88 (MyD88), and NF-κB p65 proteins in both the spinal cord and dorsal root ganglion in a spared nerve injury (SNI)-induced neuropathic pain rat model. EA also reduced the co-expression of microglial marker CD11b with TLR4/MyD88.By modulating the HMGB1/TLR4/NF-κB signaling pathway, EA effectively attenuated neuroinflammatory responses and mechanical hypersensitivity. Therefore, the anti-inflammatory effects of EA observed in these studies, specifically its downregulation of the HMGB1/NF-κB pathway, suggest its potential therapeutic value for conditions like LSS, where similar NF-κB-mediated inflammation is implicated in LF pathology.

### JAK/STAT pathway

The JAK/STAT signaling pathway, an extensively expressed intracellular signaling cascade, comprises Janus kinases (JAKs) and signal transducers and activators of transcription (STATs). Activation of this pathway occurs when cytokines or growth factors bind to their respective receptors, leading to receptor phosphorylation by JAKs and the subsequent recruitment of STAT proteins. These STAT proteins then dimerize and translocate to the nucleus, where they regulate the expression of genes involved in immune responses, cell proliferation, differentiation, and apoptosis. The JAK/STAT pathway is pivotal in inflammatory responses, as its activation modulates the expression of genes related to inflammation, thereby influencing immune modulation and cellular functions (46–48). This pathway mediates the signaling of several cytokines, including interferons (IFNs) and certain interleukins. Research has demonstrated that EA can effectively modulate inflammatory responses by influencing the JAK/STAT pathway. Evidence indicates that EA can effectively modulate inflammatory responses by targeting the JAK/STAT pathway. The anti-inflammatory effects of EA are closely linked to its ability to regulate specific receptors and downstream signaling molecules within this cascade. A compelling series of studies by Wang et al. demonstrated that in a spared nerve injury (SNI) model, 2 Hz EA treatment significantly upregulated the expression of the alpha-7 nicotinic acetylcholine receptor (α7nAChR) in the dorsal root ganglion and spinal cord. This α7nAChR activation subsequently suppressed the phosphorylation of JAK2 and STAT3, as well as rebalanced the levels of pro-inflammatory (IL-1β, IL-6) and anti-inflammatory (IL-10) cytokines (49, 50). Crucially, intrathecal administration of the α7nAChR antagonist α-bungarotoxin reversed both the analgesic effects of EA and its inhibition of JAK2/STAT3/IL-6 signaling (50), underscoring the centrality of this mechanism in EA-mediated anti-inflammatory action. This suggests that in LSS, EA might similarly attenuate local inflammation via the α7nAChR-JAK2/STAT3 axis, potentially providing a molecular basis for reducing LF thickening. Furthermore, work by YAN et al. (51) showed that EA treatment significantly downregulated the mRNA expression of Interleukin-22 (IL-22), JAK2, and STAT3, and suppressed the levels of their phosphorylated proteins (p-JAK2, p-STAT3) in the nucleus pulposus tissue of a rat model of cervical intervertebral disc degeneration (CIDD). These findings indicate that EA mitigates cell apoptosis and inflammatory responses by inhibiting the IL-22/JAK2-STAT3 signaling pathway, thereby conferring protection against intervertebral disc degeneration.

### MAPK pathway

The MAPK signaling pathway is a critical cellular signal transduction mechanism, conserved across a range of organisms, including yeast and mammals. This pathway orchestrates cellular responses to external stimuli through a series of cascade reactions and plays a role in various physiological processes such as cell growth, differentiation, proliferation, and apoptosis. It is mainly comprised of three significant subfamilies: p38 MAPK, extracellular signal-regulated kinase (ERK), and c-Jun N-terminal kinase (JNK) (52–54). These subfamilies are crucial in cell proliferation, differentiation, apoptosis, and stress responses, and also act as key mediators in inflammatory processes. Studies have shown that EA can inhibit the activation of p38 MAPK and ERK, thereby alleviating inflammation and pain. Specifically, EA suppresses the activation of p38 MAPK, which leads to a decrease in the release of inflammatory mediators. Since ERK activation is closely linked to cell proliferation and survival, EA modulates the phosphorylation state of ERK to influence inflammatory responses (53). Wu et al. demonstrated that suppressing ERK1/2 activation produces direct anti-fibrotic outcomes in LF fibroblasts, reversing the overproduction of ECM proteins that constitutes the primary scaffold of LF thickening (55). According to Fang et al. (56) demonstrated that EA effectively modulates the ERK1/2 signaling pathway in an inflammatory pain model, thereby reducing pain sensitivity. EA suppresses the activation of ERK1/2, resulting in decreased expression of inflammatory factors like COX-2 and neurokinin-1 (NK-1), which in turn affects the binding activity of the downstream transcription factor cAMP(cyclic adenosine monophosphate) response element-binding protein (CREB). In a rat model of acute inflammatory pain induced by complete Freund’s adjuvant (CFA), EA treatment elevated the mechanical paw withdrawal threshold and reduced ankle swelling, mechanistically associated with the downregulation of phosphorylated p38 MAPK (p-p38), its downstream transcription factor activating transcription factor 2 (ATF-2), and the pain receptor vanilloid receptor 1 (VR-1) in the spinal dorsal horn (57) Furthermore, in a model of pain chronification, EA demonstrated a capacity to prevent the transition to chronic pain by attenuating the expression of spinal p38 MAPK and the pivotal pro-inflammatory cytokine TNF-α (58) Critically, chronic inflammation is a well-known precursor to tissue fibrosis. As MAPK pathway activation has been established as a pivotal mechanism driving LF hypertrophy in LSS through pro-inflammatory and pro-fibrotic processes (59), we hypothesize that its targeted inhibition by EA may attenuate this structural pathology, offering a therapeutic strategy that addresses the underlying disease mechanism.

**Table 1** provides a comprehensive summary of the key preclinical studies elucidating EA’s modulation of these anti-inflammatory signaling pathways.

**Table 1:** Summary of preclinical studies on EA's anti-inflammatory signaling pathways.

Disease model	Model type	Intervention	Tissue studied	Key signaling molecules affected (Measured)	Specific cytokines measured	Reference
Knee Osteoarthritis (OA)	Rabbit model of OA induced by surgery	2/100 Hz alternating frequency EA(acupoints: ST35, EX-LE5)	Cartilage,Synovial Fluid	IKK-β ↓, IκB-α ↑, NF-κB p65 ↓	IL-1β ↓, IL-6 ↓, TNF-α ↓, MMP-3 ↓	Wu et al.(2018) (42)
Spared Nerve Injury (SNI)	Rat model of peripheral nerve injury	2 Hz EA (acupoints:ST36, SP6)	Spinal Cord	HMGB1↓, TLR4↓, MyD88↓, NF-κB p65↓, p-NF-κB p65↓	Not measured	Xia et al.(2019) (44)
Spared Nerve Injury (SNI)	Rat model of peripheral nerve injury	2 Hz EA (acupoints:ST36, SP6)	Dorsal Root Ganglion (DRG)	HMGB1↓, TLR4↓, MyD88↓, NF-κB p65↓, p-NF-κB p65↓	Not measured	Xia et al.(2024) (45)
Spared Nerve Injury (SNI)	Rat model of peripheral nerve injury	2 Hz EA (acupoints:ST36, SP6)	Dorsal Root Ganglion (DRG)	α7nAChR↑, p-JAK2↓, p-STAT3↓	IL-1β↓, IL-6↓, IL-10↑	Wang et al.(2019) (49)
Neuropathic Pain	Rat model of peripheral nerve injury	2 Hz EA (acupoints: ST36, SP6)	Spinal Cord (lumbar enlargement, L4-L6 segments)	p-JAK2↓, p-STAT3↓	IL-6↓	Wang et al.(2020) (50)
Cervical Intervertebral Disc Degeneration (CIDD)	Rat model of CIDD	2/15 Hz alternating frequencies EA (acupoints:Jiaji points)	Nucleus Pulposus tissue (C4-C6); Primary Nucleus Pulposus cells	IL-22↓, JAK2↓, STAT3↓, p-JAK2↓, p-STAT3↓, Caspase-3↓, Bax↓, Bcl-2↑	TNF-α↓, IL-6↓, IL-1β↓	Yan et al.(2024) (51)
CFA-induced chronic inflammatory pain	Rat model of inflammatory pain induced by CFA	2/100 Hz alternating frequency EA (acupoints: ST36, BL60)	Spinal Dorsal Horn (lumbar enlargement, L4-L6 segments)	p-p38 MAPK↓, p-ATF-2↓, VR-1 (TRPV1)↓,COX-2	Not measured	Fang et al.(2013) (57)
Complete Freund's Adjuvant (CFA)-induced Inflammatory Pain	Rat hindpaw CFA inflammatory pain model	2/100 Hz alternating frequency EA (acupoints: ST36, BL60)	Spinal Dorsal Horn (lumbar enlargement, L4-L6 segments)	p-ERK1/2↓ , p-Elk1, CREB↓, COX-2↓, NK-1↓	Not measured	Fang et al.(2014) (56)
Hyperalgesic Priming (HP) Model	Carrageenan/PGE_2_-induced HP model	2/100 Hz alternating frequency EA (acupoints: ST36, BL60)	Spinal Dorsal Horn (L4–6 segments)	p38 MAPK↓	TNF-α↓	Jin et al.(2021) (58)

## Clinical applications of EA in the treatment of LSS

The clinical efficacy of EA for LSS is supported by a growing body of evidence, as summarized in **Table 2**.

**Table 2:** Summary of clinical studies on EA for LSS.

Main acupoint	Adjunctive points	EA parameters (duration / stimulation frequency)	Treatment frequency & total course	Outcome measures	Conclusion	Study design	Study (year)2
Ashi points, Jiaji (EX-B2l),	Yanglingquan (GB34),Xuanzhong (GB39),Weizhong (BL40),Huantiao (GB30), Zhibian (BL54),Chengfu (BL36)Shenshu (BL23),Dachangshu (BL25)	30 minutes 2/100 Hz alternating frequency	5 sessions/wk, 4 wks (20 sessions)	Japanese Orthopaedic Association (JOA) score; MRI (herniation volume/diameter)	EA not only significantly improved clinical symptoms but might also promote remarkable resorption of the herniated lumbar disc.	Case Report	Li et al. (2020) (60)
Group 1: Jiaji points (EX-B2) at L2–L5 Group 2: Shenshu (BL23), Qihaishu (BL24), Dachangshu (BL25), Guanyuanshu (BL26)	Adjunctive points: Zhibian (BL54), Huantiao (GB30), Juliao (GB29), Yinmen (BL37), Weizhong (BL40), Yanglingquan (GB34), Ashi points, etc.	30 minutes ~1.33 Hz (80 pulses per minute)	First 3 wks: 1 session/day; Subsequent 5 wks: every other day; 8 wks total	Clinical symptom rating, total effective rate	EA combined with lumbar muscle exercise effectively relieved clinical symptoms in LSS patients with LF hypertrophy	Clinical Observation	Jia et al. (2015) (61)
Shenshu (BL23), Dachangshu (BL25), Weizhong (BL40)	Blood stasis: Geshu (BL17), Xuehai (SP10) Cold-dampness: Yaoyangguan (GV3), Mingmen (GV4) Liver-kidney deficiency: Ganshu (BL18), Shenshu (BL23), Taixi (KI3) Damp-heat: Neiting (ST44), Yinlingquan (SP9)	20 minutes (Stimulation frequency not specified in the text)	3 groups (different frequencies), 3 courses (4 wks/course)	Oswestry Disability Index (ODI); Hemorheological parameters	Daily or every-other-day EA was superior to once-every-3-days EA in improving disability and hemorheology, with the every-other-day regimen being the dominant interval.	Randomized Controlled Trial (RCT)	Xian et al. (2022) (62)
Yaoyangguan (GV3), bilateral Shenshu (BL23), Qihaishu (BL24), Dachangshu (BL25), Guanyuanshu (BL26), Huantiao (GB30), Fengshi (GB31), Weizhong (BL40), Kunlun (BL60)	Juliao (GB29),Chengfu (BL36), Yinmen (BL37),Heyang (BL55), Chengshan (BL57)	20 ± 5 minutes 3 Hz	Twice a week for 8 weeks totaling 16 sessions.	Visual Analog Scale (VAS), Self-rated Walking Distance, Short-Form McGill Pain Questionnaire (SF-MPQ), Oswestry Disability Index (ODI).	Adding acupotomy to usual care (which included acupuncture with local EA) showed no significant additive effect; the baseline regimen containing EA significantly improved symptoms over time.	Randomized Controlled Trial (RCT)	Lee et al. (2023) (63)
X-ray guided targeted nerve root (e.g., L4, L5, S1)	None	10 minutes 10 Hz	1 session/wk, 3-5 sessions total	Visual Analogue Scale (VAS) (back/leg pain, dysaesthesia), self-reported walking distance	X-ray-guided spinal nerve root EA provided rapid, significant, and sustained (for 3 months) analgesic and functional improvements in patients unresponsive to standard acupuncture.	Prospective Case Series	Inoue et al. (2012) (12)
Paraspinal muscles, gluteus medius/minimus, symptom-based peripheral nerve targets	Quadratus lumborum, piriformis muscles,Peri-neural targets including sciatic, tibial, and common peroneal nerves	20 minutes (Stimulation frequency not specified in the text)	2 sessions/wk, up to 6 wks (12 sessions)	Primary: Numeric Pain Rating Scale (NPRS), Oswestry Disability Index (ODI); Secondary: Roland Morris Disability Questionnaire (RMDI), Global Rating of Change (GROC), medication intake.	The adjunctive use of electrical dry needling and spinal manipulation provided statistically superior outcomes in pain reduction and functional improvement at the 3-month follow-up compared to conventional physical therapy alone in patients with LSS	Randomized Controlled Trial (RCT)	Young et al. (2024) (64)

Jia Xirui et al. (61) employed EA combined with lumbar and back muscle exercises to treat LSS caused by LF hypertrophy, yielding significant clinical improvements. This method alleviated lower back and leg pain, reduced numbness in the lower limbs, and enhanced functional capacity. By applying EA to the “Jiaji” acupoints (EX-B2) along the lumbar spine, the treatment diminished edema in the nerve roots and surrounding pathological tissues, strengthened lumbar and back muscle function, improved lumbar spine stability, and promoted self-repair of the LF. Ultimately, this approach relieved symptoms, enhanced lumbar stability, and elevated patients’ quality of life. Importantly, the anti-inflammatory potential of EA receives broader support from a recent systematic review and network meta-analysis, which concluded that comprehensive EA regimens (particularly EA combined with Tuina and rehabilitation) significantly reduce serum levels of pivotal pro-inflammatory cytokines, such as IL-6 and TNF-α, in patients with lumbar disc herniation (65). This suggests that the symptomatic relief and functional recovery observed in LSS patients following EA may be partly attributable to its systemic anti-inflammatory effects. A case report on lumbar disc herniation evaluated the clinical and radiological outcomes of EA monotherapy (60). Following a course of 20 EA sessions over one month, the patient’s Japanese Orthopaedic Association (JOA) score improved markedly from a baseline of 11 to 26. Crucially, MRI re-examination at the 10-month follow-up confirmed substantial resorption of the herniated mass at L4–5, with its volume reduced from 921 mm³ to 388 mm³ and the maximum anterior-posterior diameter decreasing from 11.2 mm to 5.5 mm. These findings underscore the therapeutic potential of EA in lumbar degenerative conditions and lend clinical support to the investigation of its mechanistic role in attenuating LF thickening in LSS via the modulation of inflammatory factors. Xian Peiwei et al. (62) treated 129 patients with LSS due to LF hypertrophy using varying acupuncture frequencies. Their findings suggested that more frequent acupuncture sessions were more effective in enhancing lumbar spine function. Lee et al. (63) studied 34 patients with LSS who were treated with a combination of EA, acupuncture, and interferential current therapy. The results showed significant improvements in VAS scores, self-reported walking distances, the Short-Form McGill Pain Questionnaire (SF-MPQ), and the Oswestry Disability Index(ODI), leading to substantial pain relief and symptom alleviation. In a randomized clinical trial, researchers (64) examined the efficacy of EA combined with spinal manipulation for LSS treatment. The results demonstrated that patients receiving this combination experienced significantly greater improvements in lower back, buttock, and leg pain (measured by the Numeric Pain Rating Scale(NPRS)) and disability (measured by ODI, and the Roland-Morris Disability Index(RMDI)) compared to those undergoing conventional physical therapy alone. These improvements were particularly marked at the 3-month follow-up, showing a moderate effect size. The potential mechanism of EA may involve stimulating the spinal nerve roots and modulating structures associated with nerve root compression, such as ligaments, thereby ameliorating symptoms and enhancing functional status. This study provides strong clinical evidence supporting the use of EA and spinal manipulation in the non-surgical management of LSS. Motohiro Inoue et al. (12) investigated spinal nerve root EA in 17 LSS patients unresponsive to conventional conservative treatments or traditional acupuncture. Guided by fluoroscopy, needles were positioned as close as possible to the affected nerve roots and stimulated with low-frequency EA. The results showed significant post-treatment improvements in symptoms, including lower back pain, leg pain, and sensory disturbances. These findings suggest that EA may relieve symptoms by activating pain inhibition systems and enhancing neural blood flow, offering robust clinical evidence for its role in the non-surgical treatment of LSS.

## Conclusions

As a non-surgical treatment option, EA shows promising potential in managing LSS, particularly in reducing LF hypertrophy and its associated symptoms. This approach is particularly suitable for patients reluctant to undergo surgical intervention. One of the pathological features of LSS is abnormal thickening of the LF, a process closely associated with local inflammation. Research suggests that EA may mitigate local inflammatory responses by regulating the expression of pro-inflammatory factors (such as TNF-α, IL-1β, IL-6) and anti-inflammatory factors (such as IL-10). This modulation may help to inhibit LF fibrosis and hypertrophy.

Additionally, EA may also influence key signaling pathways, including NF-κB, JAK/STAT, and MAPK, to diminish the release of inflammatory mediators and suppress inflammatory responses. Additionally, EA may retard fibrosis progression by modulating macrophage polarization. Through these multi-pathway and multi-target mechanisms, EA not only ameliorates the pathological condition of the LF but also significantly alleviates pain, enhances functional status, and improves patients’ quality of life.

In addition to regulating inflammatory factors and signaling pathways, EA may also promote local blood circulation and metabolism, enhance tissue repair capacity, and improve the elasticity and structure of the LF. These effects might be linked to its influence on ECM metabolism and the reduction of fibrosis. Clinical studies indicate that EA can be effectively combined with physical therapy and pharmacological treatments to enhance overall therapeutic efficacy, thereby further improving lumbar spine stability and function.

However, EA therapy faces several challenges in clinical practice. Firstly, individual differences can influence therapeutic efficacy, as factors such as the physician’s needling technique, the patient’s physiological traits, pathological conditions, and psychological aspects can all impact their response to EA treatment.

In patients with chronic low back pain, Toroski et al. (11) directly compared EA and NSAIDs. The EA group showed a higher health utility score (0.70 ± 0.14) than the NSAIDs group (0.63 ± 0.2), with a statistically significant difference (p ≤ 0.05). For pain intensity, the EA group also exhibited a lower VAS score (0.31 ± 0.17) compared to the NSAIDs group (0.37 ± 0.21). Notably, the EA group had a longer mean disease duration (3.4 ± 1.8 years) than the control group (2.3 ± 1.2 years). The observed advantage in utility despite a more advanced disease profile in the EA group underscores its potential clinical relevance in managing chronic low back pain. EA also enhanced autonomic nervous function and reduced sympathetic nervous activity, highlighting its comprehensive therapeutic benefits. Some may respond better to traditional treatments, underlining the influence of individual variability on EA efficacy. Furthermore, the operational techniques and treatment parameters of EA, such as needle insertion depth, frequency, and duration, have yet to be fully standardized. This lack of standardization may lead to variations in therapeutic outcomes across different clinical practices. Ian Young et al. (64) suggested that EA might not achieve optimal results in treating LSS, partly due to insufficient stimulation. Their study emphasized that appropriate needle depth and stimulation intensity are crucial for directly targeting spinal nerve roots and modulating structures associated with spinal stenosis, which could significantly enhance therapeutic efficacy. Therefore, future research should focus on optimizing EA stimulation methods and parameters to thoroughly evaluate its therapeutic effects on LSS patients. This approach would aid in improving the standardization and effectiveness of EA in clinical practice.

Although research into the mechanisms of EA therapy remains limited, existing evidence suggests that it can regulate inflammation, promote tissue repair, and enhance blood circulation through multiple pathways. Future studies should delve deeper into the specific mechanisms of EA and assess its long-term efficacy to establish a theoretical framework for its application in treating LF hypertrophy. Overall, EA appears promising as a significant adjunct in the treatment of LSS. However, its clinical application requires careful evaluation of its limitations and the optimization of treatment protocols to achieve the best therapeutic outcome.
